# BioXpress: an integrated RNA-seq-derived gene expression database for pan-cancer analysis

**DOI:** 10.1093/database/bav019

**Published:** 2015-03-28

**Authors:** Quan Wan, Hayley Dingerdissen, Yu Fan, Naila Gulzar, Yang Pan, Tsung-Jung Wu, Cheng Yan, Haichen Zhang, Raja Mazumder

**Affiliations:** ^1^Department of Biochemistry and Molecular Medicine and ^2^McCormick Genomic and Proteomic Center, The George Washington University, Washington, DC 20037, USA

## Abstract

BioXpress is a gene expression and cancer association database in which the expression levels are mapped to genes using RNA-seq data obtained from The Cancer Genome Atlas, International Cancer Genome Consortium, Expression Atlas and publications. The BioXpress database includes expression data from 64 cancer types, 6361 patients and 17 469 genes with 9513 of the genes displaying differential expression between tumor and normal samples. In addition to data directly retrieved from RNA-seq data repositories, manual biocuration of publications supplements the available cancer association annotations in the database. All cancer types are mapped to Disease Ontology terms to facilitate a uniform pan-cancer analysis. The BioXpress database is easily searched using HUGO Gene Nomenclature Committee gene symbol, UniProtKB/RefSeq accession or, alternatively, can be queried by cancer type with specified significance filters. This interface along with availability of pre-computed downloadable files containing differentially expressed genes in multiple cancers enables straightforward retrieval and display of a broad set of cancer-related genes.

**Database URL:** http://hive.biochemistry.gwu.edu/tools/bioxpress

## Introduction

Gene expression is considered a key molecular marker for diagnostic and prognostic assessment of cancer (1–8). More than a decade ago, gene expression analysis was proposed as a method to complement classification schemes based on tumor morphology because it was well known that tumors with similar histopathological appearance can have considerably different clinical outcomes (6, 9). These efforts provided the framework by which linking gene expression with cancer research could be realized (10).

Hanahan and Weinberg (11) in their seminal paper ‘The Hallmarks of Cancer’ discussed the role of over- and under-expression of key genes in several cancers. The conjectures that both diagnosis of somatically acquired lesions in tumors and genome-wide expression profiling of tumors would become routine (11) have not yet been realized, but we anticipate that this will likely change within the next decade. With advances in next-generation sequencing (NGS) technologies, several national and international projects are underway that aim to capture and analyze the expression profiles of thousands of tumors (12–14). Additionally, there are already thousands of publications that describe over- and under-expression of specific genes in cancer. Currently, to the best of our knowledge, there is no integrated view of the expression profiles of the human genes obtained from NGS technology such as RNA sequencing (RNA-seq). Moreover, no singular effort is underway to manually curate data from publications on cancer-related gene expression, enabling easy comparison of expression data and knowledge from both small publications and large-scale studies like The Cancer Genome Atlas (TCGA: http://cancergenome.nih.gov/) and International Cancer Genome Consortium (ICGC: https://icgc.org/). Lack of such efforts prevents us from tracking our knowledge of expression profiles of genes in different cancer types as technology improves and more data and information accumulate. Furthermore, as we move toward the translation of expression analysis through genomic or proteomic technologies to the clinic, there is no easy way to compare a patient’s expression data with that extant data. BioXpress has been developed as the first step toward the provision of easy access to gene expression data from tumor and normal samples, which will be useful for clinical research, diagnostics and prognostics of cancer.

The specific technology used to measure gene expression significantly affects the cost, comprehensiveness and the time consumed to perform expression analysis. DNA microarray and quantitative polymerase chain reaction (q-PCR) are powerful approaches for measuring gene expression and have been used for many years. DNA microarray technology is efficient and cost-effective at the gene expression level, while q-PCR is considered more sensitive. However, neither of these approaches can meet the sensitivity and comprehensiveness of the newer RNA-seq technology (15, 16). Despite the benefits of RNA-seq, microarrays are often preferentially used due to the higher cost and lack of standardization of pipelines using the RNA-seq technology. Once these obstacles are overcome, it is clear that RNA-seq will become the predominant tool for expression analysis (17). In addition to expression analysis, RNA-seq provides a number of other benefits. A single RNA-seq experiment output can aid in the discovery of novel and unannotated transcripts (18), single nucleotide variation (SNV) identification (19) and more (20). As RNA-seq technology and the corresponding analytical approaches grow, the application of this method is becoming indispensable for many scientific disciplines (21–23). To address this growing presence of RNA-seq data, we currently focus on large-scale integration of RNA-seq-based expression data in BioXpress complemented by manual curation of information from publications reporting gene expression associated with cancer. The manual curation process allows us to collect valuable expression-related information from peer-reviewed publications from diverse platforms. Integration of information from both large-scale studies and publications allows users to easily compare and contrast expression profiles of their gene(s) of interest.

The advancement of expression analysis technology has led to the development of corresponding databases and standards. For example, the Minimum Information About a Microarray Dataset initiative (24) provides standards for microarray data, while databases like the National Center for Biotechnology Information (NCBI) Gene Expression Omnibus (GEO) (25) and Array Express (26) have significant amounts of microarray data. Secondary databases that store and provide results and analysis of microarray and other gene expression data related to cancer such as CGED (Cancer Gene Expression Database) (27), GENT (Gene Expression across Normal and Tumor tissue) (28) and Oncomine (29) are also available. Finally, TCGA and ICGC data portals and databases, such as Expression Atlas (30), provide RNA-seq-generated data. All the above-mentioned databases provide mechanisms to retrieve gene-specific information, but, to the best of our knowledge, none of them allows integrated pan-cancer analysis across multiple projects. NCBI GEO and European Bioinformatics Institute (EBI) ArrayExpress, e.g. are public repositories for high-throughput microarray and NGS functional genomic datasets. A gene symbol-based search can result in thousands of profiles from GEO Profiles Database. CGED, on the other hand, provides data specifically obtained through collaborative efforts of Nara Institute of Science and Technology, Osaka University Medical School, Kyoto University Medical School and Osaka Medical Center for Cancer and Cardiovascular Diseases. GENT provides Affymetrix microarray data from tumor and normal samples, while Expression Atlas at EBI provides differential and baseline expression from several organisms. Similar to other public repository data, a single search can retrieve data from many experiments in these resources. Furthermore, although tools do exist which aim to analyze the same scope of data, the tools and databases of which we are aware do not facilitate the expression analysis on RNA-seq desired here. cBioPortal (31) is a widely popular resource with an emphasis on mutation analysis. Currently, Oncomine (29) does provide the means to analyze expression for microarray data, but not for RNA-seq. Thus, there is no single tool/resource available which integrates RNA-seq information that allows expression analysis to identify, e.g. cancer relatedness. Although hundreds of cancer RNA-seq studies are published each year, a cancer-centric RNA-seq expression database that integrates all cancer-related RNA-seq-based expression data from databases and publications is not available to the community. Portals like TCGA data portal and ICGC data portal, which provide RNA-seq-based expression data, only provide access to raw read counts and normalized counts: such data cannot be easily used for comparative analysis across several cancer types and existing experimental results in publications. In addition, different normalization methods are employed by different data providers, making comparison and cross-type analysis even more challenging.

The BioXpress database project collects RNA-seq data from several publicly available sources such as TCGA (http://cancergenome.nih.gov/), ICGC (12) and Gene Expression Atlas (30), and uses a standardized method to identify the expression levels of the genes. Expression levels of genes are also manually extracted from publications to supplement information gathered from large-scale studies. Additionally, all cancer types are mapped to Disease Ontology (32) terms to facilitate pan-cancer analysis. Finally, all genes are linked to a comprehensive cancer-related non-synonymous SNV database, BioMuta (33). Together, BioMuta and BioXpress provide a detailed view of the expression and mutations of genes in cancer and therefore can be used for pan-cancer studies like the one performed by our group recently (34) and described in this manuscript.

## Data Source and Metrics

**Figure 1.**
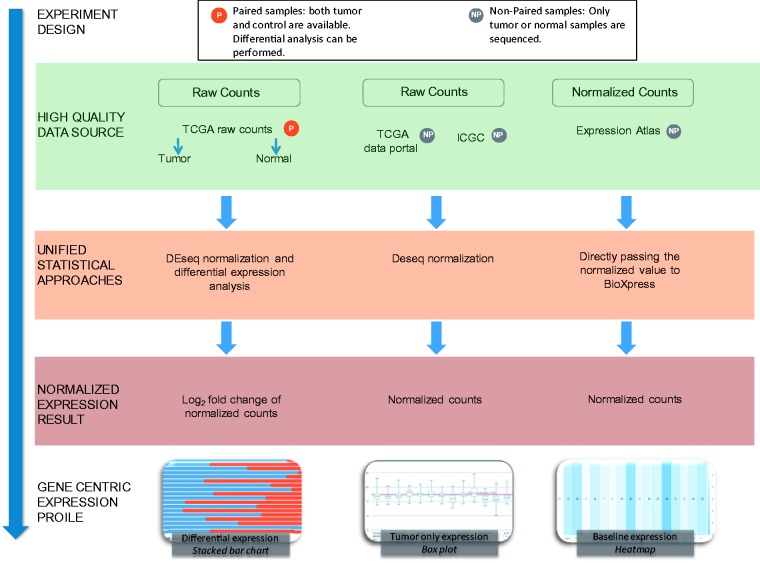
Flow chart of the workflow used to create BioXpress. BioXpress processes short reads and read count data through distinct pipelines. Data are further divided into two groups: paired data that have both normal and tumor samples from the same patient, and non-paired, tumor-only data. Output in BioXpress is split into three different types: differential expression (stacked bar chart), tumor-only expression (box plot) and baseline expression data (heatmap). In addition to the data integration approaches shown in the figure, gene expression information is also extracted from publications.

The majority of RNA-seq databases provide data either in FASTQ format (sequence reads) and/or raw read count data. Read count data are calculated by analyzing the mapping file where the reads have already been aligned to a reference genome. As shown in Figure 1, BioXpress processes data based on the availability of expression data from paired data that have both normal and tumor samples from the same patient, and the non-paired data from just tumor and also from just normal tissue.

The data sources and statistics in terms of number of patients from each data source are shown in Table 1. To achieve comprehensiveness, data are collected from TCGA, the Curated Short Read archive (CSR) (35), ICGC (12), Gene Expression Atlas (30) and publications (Table 1). It is important to note that ICGC, at the time of writing this article, did not contain any data from paired normal and tumor samples which are not from TCGA. Therefore, the data in BioXpress are split into three different types: differential expression, tumor-only expression and baseline expression data from Illumina Human Body Map project (http://www.ebi.ac.uk/gxa/experiments/E-MTAB-513).

**Table 1.** Statistics of data collected in BioXpress

Source	Data type	No. of samples/individuals^a^	Tumor/normal
TCGA	Raw read count	1320/660^b^	Tumor and normal
ICGC and TCGA	Raw read count	6397/6324	Tumor
Expression Atlas baseline	Normalized count	1/1	Normal
Literature	Published literature	Not applicable (135 publications)	Tumor and normal comparison

^a^Typically, each patient contains more than one sequencing sample. Therefore, we provide the number of both samples and individuals.

^b^The number of patients is collected from TCGA, ICGC and Expression Atlas baseline projects. Some TCGA patient IDs overlap with the ICGC patient IDs.

## Data Processing

### TCGA data portal

TCGA-Assembler was used to download RNA-seq data from TCGA data portal. Raw counts data with paired samples (tumor and normal) were extracted and analyzed using DEseq R package with default parameters: method = ‘blind’, sharingMode = ‘fit-only’, fitType = ‘local’ (36). DEseq normalization method has been reported to outperform other normalization methods (37). Fold changes, not absolute expression values, are displayed based on analysis described above (38). False discovery rates are not defined due to the low number of replicates for samples. This approach allows the user to determine the significance of differentially expressed genes on an individual basis.

### ICGC data portal

ICGC contains tumor-only data (normal samples are not sequenced by the consortium currently). Gene expression data from tumor samples was downloaded from ICGC data portal (12) and analyzed using DEseq R package with default parameters (36).

### Expression atlas

Normalized baseline expression was downloaded via Expression Atlas (http://www.ebi.ac.uk/gxa/download.html) (30). Because raw read counts are not available for all data retrieved from Expression Atlas, no additional normalization was performed in BioXpress.

### Manual curation from publications

Decades of research on differential expression in tumor and normal samples has led to thousands of publications. Although many of these studies are based on samples from modest numbers of patients, there is value in the systematic capture and presentation of this information alongside large-scale studies such as those presented by TCGA and ICGC. Although it is possible that studies may exhibit discordance, it is equally possible for the consideration of such additional experiments to contribute to the ‘big picture’ of differential expression between tumor and normal samples. We leave it to the discretion of individual users to decide the significance of curated publications in application to their studies.

For manual curation of expression data, genes identified in our previous pan-cancer study were prioritized (34). In addition to this prioritization, genes annotated by UniProtKB/Swiss-Prot as associated with cancer and Cancer Gene Census (http://www.sanger.ac.uk/genetics/CGP/Census/) (39) were also targeted for manual curation. This UniProtKB/Swiss-Prot gene list was obtained using the following search string: organism: ‘Homo sapiens [9606]’ AND reviewed:yes AND annotation:(type:disease cancer). Briefly, the manual curation protocol involved searching PubMed (40) using the gene name (including synonyms) with accompanying text ‘cancer’ and ‘expression’. The curator then reviewed the title to shortlist articles which appear to contain gene expression information related to cancer and have full text available. Abstracts were then read to identify potential true positive articles. All such articles were downloaded and read to extract key information such as cancer type and expression information. All cancer types were then mapped to Disease Ontology terms (32) and added to the BioXpress database. To date, 536 papers have been filtered to maintain only those focusing on human cancer after reading the ‘Abstract’ and ‘Introduction’. Among this subset, only papers including direct evidence reflecting gene expression differentiation between normal and cancer tissues were kept. Filtering then continued with further inspection of the ‘Materials and Method’ and ‘Results’ sections of each paper. Some cancer-type abbreviations were taken from the TCGA Code Table Report (https://tcga-data.nci.nih.gov/datareports/codeTablesReport.htm), while the rest of them were named using the following conventions: first three letters from the first word and the last two letters from the second word. Thus, if the cancer types have a single word name, all five letters come from this word. In the event of duplication, letters from the third or fourth words are used to distinguish between types. Curators cross-check all manual curation processes. In total, 135 papers concerning 87 genes have been added to the BioXpress database through biocuration (supplementary Table S2).

### Data Normalization and Analysis

DEseq method is regarded as one of the most robust RNA-seq normalization methods (37). In the BioXpress pipeline, raw counts data were normalized by DEseq method followed by differentially expressed gene analysis. To compare non-paired samples with normalized results from DEseq pipeline, the DEseq normalization method was used [Parameters: library(‘DESeq’), cds = newCountDataSet(data,condition), cds = estimateSizeFactors(cds), result = counts(cds,normalized = TRUE)]. For differential expression analysis, gene expression was normalized based on each patient, and case and control were considered together. For tumor expression, all samples were collectively analyzed across different cancer types and then normalized. Heat map and clustering analysis were performed using the ‘heatmap’ function from the R package (http://www.R-project.org/).

## Usage and Utility

Scientists can find querying datasets useful to identify expression levels between disease and normal pairs to discover differential expression for a gene. They may also want to research on potential biomarkers or pathways that lead to tumor formation or want to explore the overall expression of specific genes across multiple cancer types. Users can search BioXpress using HGNC-approved gene symbols (HUGO Gene Nomenclature Committee), UniProtKB/Swiss-Prot accessions or RefSeq accessions. Differentially expressed genes for a specific cancer type can also be retrieved. Additionally, all data in BioXpress, including lists of genes significantly differentially expressed in two or more cancer types, can be downloaded.

### Searching using gene name (gene/protein-centric search)

A search using the HGNC-approved gene symbol or UniProt/RefSeq accession retrieves differential expression information (cancer vs. normal), tumor-only expression data (where normal samples are not available) and baseline expression information from normal human tissues (Illumina Human Body Map Project). The example below provides an overview of a gene/protein-centric search.

#### Differential expression

**Figure 2.**
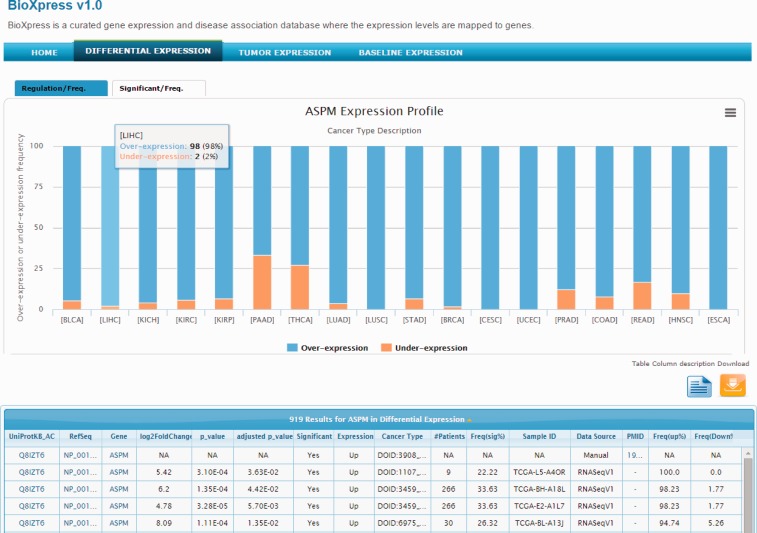
Snapshot of BioXpress interface. The stacked bar chart displays the percent of individuals with over- or under- expression of the ASPM gene.

The abnormal spindle-like microcephaly-associated (ASPM) gene is highly expressed in several tumor cell lines (41) and cancers (42, 43). Searching the BioXpress database using the gene ASPM users can retrieve the differential expression profile of this gene in different cancers. For ASPM gene, we can clearly see that this gene appears to be over-expressed in almost all cancers. Figure 2 provides a view of the BioXpress interface where the Differential Expression tab on the top menu bar is selected, and below it ‘ASPM Expression Profile’ is shown. The default view provides expression frequency (over- or under-expression) in the patients. The number of patients for a particular cancer type, *P* value and a variety of additional information is available in the table below which can be downloaded. Full cancer names are available on clicking the cancer abbreviations in figure and additional details about the data can be viewed by clicking the ‘Table column description’ link. All columns can be sorted and users can send an e-mail to the help desk with comments about a specific data element by clicking on the envelope link available from each row.

The tab at the top of the stacked bar chart provides an alternate view where users can see the frequency (number of patients) of significantly over- or under-expressed genes (based on a *P* value cutoff of 0.05). For ASPM, on clicking the Significant/Freq tab, we can see that this gene is significantly over-expressed in more than 25% of the patients in several cancers. For example, ASPM is over-expressed in breast invasive carcinoma (DOID:3459; 113 patients), lung adenocarcinoma (DOID:3907; 50 patients) and others. Combining the stacked bar frequency expression (Regulation/Freq) and the Significant/Freq, users can get a complete overview of the differential expression of a gene in all cancer types in the database.

#### Tumor expression

Clicking on the Tumor Expression tab on the top menu bar shows the expression profile for the ASPM gene from all patient samples without paired normal data. Although ICGC does not currently collect any paired data, tumor-only expression data can provide an overview of the expression of a specific gene in different cancer types and can be used in conjunction with differential and baseline expression data to better understand the comprehensive expression profile of a gene. The box plot provides the minimum lower quartile, median upper quartile and maximum expression value, and therefore provides a snapshot of the distribution of expression of a gene in all patients with a specific cancer. For the ASPM gene, we see that for cervical squamous cell carcinoma (CESC), the minimum, maximum and the lower and upper quartile are above the theoretical mean for all cancer types which could indicate that for CESC this gene has less variability in terms of expression in the patients and is expressed at a higher level compared with other cancers. Therefore, the box plot allows the user to identify cancer types where the lower and the upper quartile are short, signifying homogeneity in the expression of the gene for that specific cancer. The table below the box plot provides details such as UniProtKB accession, RefSeq accession and number of samples.

#### Baseline expression

Clicking the Baseline Expression tab for ASPM gene shows the heatmap with testis being the only tissue with increased expression of ASPM. It has been known for some time that ASPM is over-expressed in testis (41, 44), although the precise function of this gene in testis development is still unknown (45).

### Searching using cancer type (cancer type centric search)

Users may want to retrieve a list of genes that are significantly differentially expressed in a specific cancer. From the Home page, clicking on the Search by cancer type tab allows users to select the cancer type of interest and then retrieve genes which are either over- or under-expressed. For example, selecting lung adenocarcinoma and the default settings (over-expressed; adjusted *P* value and *P* > 0.1) retrieves the 2089 genes, out of which the top expressed gene is FAM83A (Protein FAM83A; also called Tumor antigen BJ-TSA-9). It is interesting to note that FAM83A is considered a promising tumor biomarker of lung cancer (41). Similarly, the second highly expressed gene GREM1 (Gremlin) is also known to be over-expressed in lung cancer (46).

### Pan-cancer analysis

**Figure 3.**
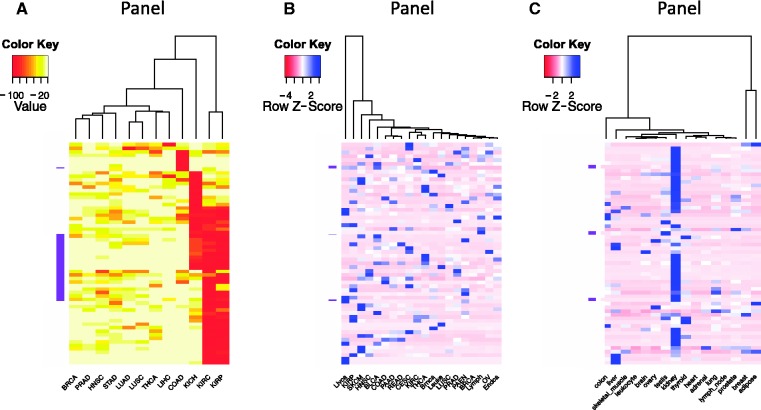
Clustering and heatmap view of the top 50 differentially expressed genes as reported by BioXpress. Although these graphics were generated using external tools, the emphasis here is the ability of BioXpress to sort through large amounts of data and return candidate subsets for subsequent analysis. (**A**) Clustering of these genes in different cancer types based on the frequency of patients who have significant differential expression. Darker colors indicate a higher percentage of patients with such differential expression. (B) For genes which do not have normal samples, the heatmap shows clustering based on normalized count. Darker colors indicate a higher expression level. (**C**) Clustering based on baseline expression for the 50 genes in different tissues. Darker colors indicate higher expression level.

The ability to sort, filter and further analyze the gene expression data collected in BioXpress allows users to compare and contrast expression of genes across many patients and cancer types. In addition to listing the genes that are significantly differentially expressed in multiple cancers (as described in the previous paragraph), Figure 3 provides an overview of the types of analysis that users can perform using the downloaded data. Figure 3A heatmap and clustering were performed based on the percent of patients who have significantly differentially expressed genes. Clustering of samples or datasets across multiple cancer types, known as one type of pan-cancer analysis, is widely conducted by the community, especially by TCGA Research Network (47, 48), and is of great interest from the aspect of personalized and translational medicine. To select genes that have strong association with transcriptomic changes of tumors, we picked the top 50 genes that are differentially expressed in the highest percent of samples. The darker colors in the figure show that several cancer types have genes which are differentially expressed in a majority of the patients (red boxes). The clustering based on the heatmap indicates that several cancer types have similar patterns [kidney renal clear cell carcinoma (KIRC) and kidney renal papillary cell carcinoma (KIRP); head and neck squamous cell carcinoma (HNSC) and stomach adenocarcinoma (STAD); lung squamous cell carcinoma (LUSC) and pancreatic adenocarcinoma (PAAD); thyroid carcinoma (THCA) and lung adenocarcinoma (LUAD)]. Figure 3B shows analysis results of expression data where no normal samples are available. The figure provides a view of cancer types that cluster together based on gene expression from cancer samples only. On the basis of the color distribution, it can be seen that several cancers have similar expression patterns and hence cluster together: breast cancer (BRCA) and lymphoma (Lymph); ovarian cancer (OV) and endometrial cancer (Endca); close to them are endocrine pancreas cancer (PAEN), prostate adenocarcinoma (PRAD), lung squamous cell carcinoma (LUSC), leukemia (Leuke) and brain cancer (Braca); KIRC and THCA; colon adenocarcinoma (COAD), PAAD and rectum adenocarcinoma (READ) are also clustered. Liver cancer (Livca) shows a distinct gene expression profile with all other cancer types listed based on the selected genes. Figure 3C provides a view of tissues which have similar expression patterns.

Collection of expression data from multiple cancers as presented in supplementary Table S1 allow us to identify genes that are differentially expressed in more than one cancer type. For example, from this table we can see that nine genes are differentially expressed in all cancer types (Table 2). It is important to note that in this particular case we do not consider the number of patients who have these genes over- or under-expressed. Therefore, each gene and its expression in a cancer type needs to be carefully evaluated on a case-by-case basis if one is interested in identifying genes which are differentially expressed in majority of the patients (please see examples in the next paragraph). It is interesting to note that five of the nine proteins are glycoproteins, two are phosphoproteins, six of them are secreted and seven are involved in biological process regulation (based on UniProtKB keyword and Gene Ontology annotation). This type of filtering and sorting can reveal ideal candidates for further evaluation as diagnostic or therapeutic targets. Furthermore, literature evidence reveals that eight of the 9 genes in Table 2 are genes known to be associated with cancer. For example, the first gene listed in Table 2, CCL21, participates in leukocytes and cancer cell migration through the CCR7/CCL19 (CCL21) axis to promote the growth and metastasis of various tumors such as breast cancer, melanoma, non-small cell lung cancer, head and neck, gastrointestinal and hematologic cancer (49). Second, γ-glutamyltransferase is involved in cellular glutathione homeostasis, its expression is often significantly increased in human tumors and its role in tumor progression, invasion and drug resistance has been repeatedly suggested (50). Third, alterations in the ubiquitin system have direct or indirect roles in the genesis of various tumors due to defects in the ubiquitin-dependent proteolysis of critical house-keeping genes or cell–cycle elements—p53 is a good example (51). The next genes, Matrilysin (MMP7), are frequently over-expressed in human cancer tissues and are associated with cancer progression (52) and NCAM1 has been demonstrated to be one of the immunohistochemical markers for lung neuroendocrine tumors diagnosis (53), its expression level is up-regulated in large cell lung tumor cell line H460-M (54). CHRDL1 is down-regulated (79–89% of 19) in follicular thyroid carcinoma (55) and the gene, WFDC2 (HE4), contains dispersed evidence: it has been demonstrated to be a biomarker for ovarian carcinoma (56) and it is known to be over-expressed in a range of different cell lines including ovarian, renal, lung, colon and breast lines, and cancers such as endometrial adenocarcinomas (57, 58) and lung adenocarcinoma (59). The next gene, LCN2, has a wide range of functions in different types of cancers (thyroid, pancreatic, breast and colon cancer), and it is a potential diagnostic and prognostic marker in both benign and malignant human diseases (60). Finally, KRT80 and its role in cancer is not well studied although there is some evidence that this gene is differentially expressed in certain types of cancer (61, 62). In addition to this list, a separate, pre-computed table which lists all genes and their normalized expression values in tumors across all cancer types is also provided for download. This table can be used to identify genes which have, e.g. high variability in expression in certain cancers or low variability (possible house-keeping genes).

**Table 2.** Genes significantly differentially expressed in tumor and normal samples in all cancer types in one or more patients

Gene	UniProtKB AC	Protein name	Over-expressed cancer types	Under-expressed cancer types
CCL21	O00585	C-C motif chemokine 21	KIRC, LIHC, BRCA, THCA, KICH	KICH, BRCA, THCA, PAAD, ESCA, KIRC, COAD, KIRP, STAD, CESC, LIHC, HNSC, READ, PRAD, BLCA, LUAD, LUSC, UCEC
GGT6	Q6P531	γ-glutamyltransferase 6	BRCA,THCA, PAAD, BLCA, STAD, CESC, LIHC, KIRC, LUAD, UCEC	BLCA, BRCA, STAD, ESCA, KIRC, COAD, KIRP, HNSC, READ, PRAD, KICH, LUAD, LUSC
UBD	O15205	Ubiquitin D	KICH, BRCA, THCA, ESCA, KIRC, COAD, STAD, CESC, LIHC, HNSC, READ, PRAD, BLCA, LUAD, LUSC, UCEC	BRCA, THCA, PAAD, KICH, KIRP, LIHC, HNSC, PRAD, BLCA
MMP7	P09237	Matrilysin	BRCA, STAD, THCA, ESCA, BLCA, COAD, PAAD, LIHC, HNSC, READ, PRAD, KIRC, LUAD, LUSC, UCEC	KICH, BRCA, BLCA, KIRP, CESC, LIHC, HNSC, PRAD, KIRC, LUAD
NCAM1	P13591	Neural cell adhesion molecule 1	BRCA, THCA, KIRC, KIRP, HNSC, KICH, LUAD, LUSC	KICH, BRCA, STAD, KIRP, THCA, ESCA, KIRC, COAD, PAAD, CESC, LIHC, HNSC, READ, PRAD, BLCA, UCEC
CHRDL1	Q9BU40	Chordin-like protein 1	PRAD, KICH, LIHC, THCA, KIRC	PAAD, BRCA, STAD, THCA, ESCA, BLCA, COAD, KIRP, KIRC, CESC, LIHC, HNSC, READ, PRAD, KICH, LUAD, LUSC, UCEC
WFDC2	Q14508	WAP four-disulfide core domain protein 2	BRCA, STAD, PAAD, ESCA, KIRC, CESC, LIHC, HNSC, BLCA, LUAD, UCEC	KICH, BRCA, THCA, BLCA, COAD, KIRP, STAD, LIHC, HNSC, READ, PRAD, KIRC, LUAD, LUSC
LCN2	P80188	Neutrophil gelatinase-associated lipocalin	BLCA, BRCA, THCA, PAAD, ESCA, KIRC, COAD, KIRP, STAD, CESC, LIHC, READ, PRAD, KICH, LUAD, LUSC, UCEC	BRCA, THCA, KIRC, KIRP, LIHC, HNSC, PRAD, BLCA, LUAD, LUSC
KRT80	Q6KB66	Keratin, type II cytoskeletal 80	BRCA, THCA, PAAD, ESCA, BLCA, COAD, KIRP, STAD, CESC, LIHC, READ, PRAD, LUAD, LUSC, UCEC	BLCA, BRCA, THCA, KIRC, LIHC, HNSC, PRAD, KICH

LIHC = liver hepatocellular carcinoma; BLCA = bladder urothelial carcinoma; KICH = kidney chromophobe; UCEC = uterine corpus endometrial carcinoma; ESCA = esophageal carcinoma; CESC = cervical squamous cell carcinoma and endocervical adenocarcinoma.

As mentioned above, one of the key questions in pan-cancer analysis of gene expression is—are there any genes which are significantly over- or under-expressed in multiple cancers in a large number of the patients. Supplementary Tables S3 and S4 provide the list of genes that are significantly differentially expressed in greater than 30% and 50% of the patients. Table 3 lists the top 5 genes (sorted based on the number of cancer types it is differentially expressed in) that are significantly over- and under-expressed in more than 50% of the patients. The first gene COL11A1 is known to be over-expressed in various epithelial cancers and is prominently correlated with invasion and metastasis (63). Its over-expression is associated with colorectal cancer (64), non-small cell lung cancer (65) and several other cancers (66). The next gene MMP11 over-expression is correlated with the aggression and invasion status of various types of carcinoma and is almost absent in normal adult organs and can be considered as a biomarker for diagnosis and prognosis (67, 68). TMPRSS4 is highly expressed in pancreatic, colon, lung and gastric cancers, and is also expressed in a wide range of human cancer cell lines and has been demonstrated to facilitate the invasion, migration and metastasis of tumor cells (69, 70). MMP1 is highly expressed in gastric carcinoma, breast cancer, lung and other cancers (71–78). ADH1B is the first gene in the table that is known to be under-expressed in multiple cancers such as oral tongue squamous cell carcinoma (79) and intrahepatic cholangiocarcinoma (80). MT1H is under-expressed in adenoid cystic carcinoma of salivary gland, prostate and liver cancer due to hypermethylation of its promoter (81, 82). In the next gene MT1G, the promoter is hypermethylated which results in its down-regulation in hepatoblastoma and prostate cancer (83, 84). CHRDL1 interestingly is under-expressed in colorectal cancer (85) while over-expressed in pancreatic cancer (86) and for CA4 there is currently no publication associated with expression of these gene in cancers. We believe that filtering and sorting of data in BioXpress will help researchers to focus on expression profiles of genes which currently have very little published information. Another gene SFRP1 which is also found to be under-expressed in our dataset in five cancers (>50% of the patients) is known to be under-expressed in nine cancer types: cancers of the kidney, stomach, small intestine, pancreas, parathyroid, adrenal gland, gall bladder, endometrium, renal cell carcinoma and testis (87).

**Table 3.** Top five genes significantly differentially expressed in tumor and normal samples in >50% of the patients

Gene	UniProtKB AC	Protein name	Over-expressed cancer types	Under-expressed cancer types
COL10A1	Q03692	Collagen alpha-1(X) chain	BRCA, STAD, BLCA, COAD, HNSC, LUAD	
COL11A1	P12107	Collagen alpha-1(XI) chain	BRCA, COAD, HNSC, LUAD, LUSC,	
MMP11	P24347	Stromelysin-3	BRCA, BLCA, COAD, HNSC, LUAD	
TMPRSS4	Q9NRS4	Transmembrane protease serine 4	KIRC, LUAD, LUSC, THCA, UCEC	
MMP1	P03956	Interstitial collagenase	COAD, LUAD, LUSC, HNSC	
ADH1B	P00325	Alcohol dehydrogenase 1B		BLCA, THCA, KIRC, COAD, KIRP, HNSC, KICH, LUSC, UCEC
MT1H	P80294	Metallothionein-1H		KICH, KIRC, KIRP, LIHC, THCA
MT1G	P13640	Metallothionein-1G		KICH, KIRC, KIRP, LIHC, THCA
CHRDL1	Q9BU40	Chordin-like protein 1		BLCA, KICH, KIRC, THCA, UCEC
CA4	P22748	Carbonic anhydrase 4		BRCA, COAD, KIRP, LUAD, LUSC

The genes were sorted based on the number of cancer types they were differentially expressed in.

LIHC = liver hepatocellular carcinoma; BLCA = bladder urothelial carcinoma; KICH = kidney chromophobe; CESC = cervical squamous cell carcinoma and endocervical adenocarcinoma.

### Downloadable files

Websites are ideal for performing gene and cancer-centric searches as described above. Some users may wish to perform large-scale analysis or filter the data based on additional parameters. To accommodate such users, all data can be downloaded in tab-delimited format. Additionally, a table of significantly under- or over-expressed genes in one or more patients is provided that has the following columns: gene name, UniProtKB accession, protein name, cancer types where the gene is expressed and count of the number of cancer types (supplementary Table S1). This table can be used to quickly identify genes that are differentially expressed in multiple cancer types in one or more patients. Additional downloads include PubMed Identifiers (PMIDs) and accessions that were manually curated (supplementary Table S2) and all data associated with differential and tumor-only expression. Future plans include addition of additional tables based on user requests.

## Future Perspective

BioXpress will be updated every 6 months and detailed statistics for each release will be provided. Such statistics will allow users to track changes in the database over time. We will also integrate BioXpress in the High-performance Integrated Virtual Environment (HIVE) NGS and proteomics analysis platform. This integration will allow users to upload RNA-seq data, map reads to the reference genome using HIVE Hexagon (88), perform expression analysis and directly compare results with those available from BioXpress. As proteomic data become available for different cancer types through programs similar to the Clinical Proteomic Tumor Analysis Consortium (CPTAC) (89), we will map such data to the genes. We also plan to augment both data and function based on input from our users. Some potential new features include the following: addition of cancer subtypes; linking BioXpress to BioMuta (33) to obtain comprehensive view of expression as it may relate to mutation; integration of clinical annotations; inclusion of additional graphical elements and more. Our preliminary results show that there is a correlation between mutation density of a gene and its expression in certain types of cancer. We intend to explore this further in our future studies.

## Supplementary Data

Supplementary data are available at *Database* Online.

## References

[1] Sotiriou C. Piccart M.J. (2007) Taking gene-expression profiling to the clinic: when will molecular signatures become relevant to patient care? *Nat**.* Rev. Cancer, 7, 545–553. 10.1038/nrc2173 17585334

[2] Normanno N. De Luca A. Carotenuto P. (2009) Prognostic applications of gene expression signatures in breast cancer. Oncology, 77(Suppl. 1), 2–8. 20130425 10.1159/000258489

[3] Mehta S. Shelling A. Muthukaruppan A. (2010) Predictive and prognostic molecular markers for cancer medicine. Ther. Adv. Med. Oncol., 2, 125–148. 21789130 10.1177/1758834009360519 PMC3126011

[4] van't Veer L.J. Bernards R. (2008) Enabling personalized cancer medicine through analysis of gene-expression patterns. Nature, 452, 564–570. 18385730 10.1038/nature06915

[5] van 't Veer L.J. Dai H. van de Vijver M.J.*.* (2002) Gene expression profiling predicts clinical outcome of breast cancer. Nature, 415, 530–536. 11823860 10.1038/415530a

[6] Golub T.R. Slonim D.K. Tamayo P.*.* (1999) Molecular classification of cancer: class discovery and class prediction by gene expression monitoring. Science, 286, 531–537. 10521349 10.1126/science.286.5439.531

[7] Wang Y. Klijn J.G. Zhang Y.*.* (2005) Gene-expression profiles to predict distant metastasis of lymph-node-negative primary breast cancer. Lancet, 365, 671–679. 15721472 10.1016/S0140-6736(05)17947-1

[8] Ntzani E.E. Ioannidis J.P. (2003) Predictive ability of DNA microarrays for cancer outcomes and correlates: an empirical assessment. Lancet, 362, 1439–1444. 14602436 10.1016/S0140-6736(03)14686-7

[9] Chung C.H. Bernard P.S. Perou C.M. (2002) Molecular portraits and the family tree of cancer. Nat. Genet., 32(Suppl), 533–540. 12454650 10.1038/ng1038

[10] Editorial. (2002) Gene expression and cancer: getting it together. Nat. Genet., 31, 1–2. 11984554 10.1038/ng0502-1

[11] Hanahan D. Weinberg R.A. (2000) The hallmarks of cancer. Cell, 100, 57–70. 10647931 10.1016/s0092-8674(00)81683-9

[12] Zhang J. Baran J. Cros A.*.* (2011) International Cancer Genome Consortium Data Portal—a one-stop shop for cancer genomics data. Database (Oxford), 2011, bar026. 21930502 10.1093/database/bar026 PMC3263593

[13] Hoadley K.A. Yau C. Wolf D.M.*.* (2014) Multiplatform analysis of 12 cancer types reveals molecular classification within and across tissues of origin. Cell, 158, 929–944. 25109877 10.1016/j.cell.2014.06.049 PMC4152462

[14] Hudson T.J. Anderson W. Artez A.*.* (2010) International network of cancer genome projects. Nature, 464, 993–998. 20393554 10.1038/nature08987 PMC2902243

[15] Shendure J. (2008) The beginning of the end for microarrays? *Nat**.* Methods, 5, 585–587. 10.1038/nmeth0708-585 18587314

[16] Mortazavi A. Williams B.A. McCue K. (2008) Mapping and quantifying mammalian transcriptomes by RNA-Seq. Nat. Methods, 5, 621–628. 18516045 10.1038/nmeth.1226 PMC13303166

[17] Zhao S. Fung-Leung W.P. Bittner A. (2014) Comparison of RNA-Seq and microarray in transcriptome profiling of activated T cells. PLoS One, 9, e78644. 24454679 10.1371/journal.pone.0078644 PMC3894192

[18] Haas B.J. Zody M.C. (2010) Advancing RNA-Seq analysis. Nat. Biotechnol., 28, 421–423. 20458303 10.1038/nbt0510-421

[19] Quinn E.M. Cormican P. Kenny E.M. (2013) Development of strategies for SNP detection in RNA-seq data: application to lymphoblastoid cell lines and evaluation using 1000 genomes data. PLoS One, 8, e58815. 23555596 10.1371/journal.pone.0058815 PMC3608647

[20] McGettigan P.A. (2013) Transcriptomics in the RNA-seq era. Curr. Opin. Chem. Biol., 17, 4–11. 23290152 10.1016/j.cbpa.2012.12.008

[21] Saliba A.E. Westermann A.J. Gorski S.A. (2014) Single-cell RNA-seq: advances and future challenges. Nucleic Acids Res., 42, 8845–8860. 25053837 10.1093/nar/gku555 PMC4132710

[22] Miller A.C. Obholzer N.D. Shah A.N. (2013) RNA-seq-based mapping and candidate identification of mutations from forward genetic screens. Genome Res., 23, 679–686. 23299976 10.1101/gr.147322.112 PMC3613584

[23] Soon W.W. Hariharan M. Snyder M.P. (2013) High-throughput sequencing for biology and medicine. Mol. Syst. Biol., 9, 640. 23340846 10.1038/msb.2012.61 PMC3564260

[24] Brazma A. Hingamp P. Quackenbush J.*.* (2001) Minimum information about a microarray experiment (MIAME)-toward standards for microarray data. Nat. Genet., 29, 365–371. 11726920 10.1038/ng1201-365

[25] Barrett T. Wilhite S.E. Ledoux P.*.* (2013) NCBI GEO: archive for functional genomics data sets—update. Nucleic Acids Res., 41, D991–D995. 23193258 10.1093/nar/gks1193 PMC3531084

[26] Parkinson H. Sarkans U. Kolesnikov N.*.* (2011) ArrayExpress update—an archive of microarray and high-throughput sequencing-based functional genomics experiments. Nucleic Acids Res., 39, D1002–D1004. 21071405 10.1093/nar/gkq1040 PMC3013660

[27] Kato K. Yamashita R. Matoba R. (2005) Cancer gene expression database (CGED): a database for gene expression profiling with accompanying clinical information of human cancer tissues. Nucleic Acids Res., 33, D533–D536. 15608255 10.1093/nar/gki117 PMC540071

[28] Shin G. Kang T.W. Yang S. (2011) GENT: gene expression database of normal and tumor tissues. Cancer Inform., 10, 149–157. 21695066 10.4137/CIN.S7226 PMC3118449

[29] Rhodes D.R. Kalyana-Sundaram S. Mahavisno V.*.* (2007) Oncomine 3.0: genes, pathways, and networks in a collection of 18,000 cancer gene expression profiles. Neoplasia, 9, 166–180. 17356713 10.1593/neo.07112 PMC1813932

[30] Kapushesky M. Emam I. Holloway E. (2010) Gene expression atlas at the European bioinformatics institute. Nucleic Acids Res., 38, D690–D698. 19906730 10.1093/nar/gkp936 PMC2808905

[31] Gao J. Aksoy B.A. Dogrusoz U.*.* (2013) Integrative analysis of complex cancer genomics and clinical profiles using the cBioPortal. Sci. Signal., 6, pl1. 23550210 10.1126/scisignal.2004088 PMC4160307

[32] Schriml L.M. Arze C. Nadendla S. (2012) Disease ontology: a backbone for disease semantic integration. Nucleic Acids Res., 40, D940–D946. 22080554 10.1093/nar/gkr972 PMC3245088

[33] Wu T.J. Shamsaddini A. Pan Y. (2014) A framework for organizing cancer-related variations from existing databases, publications and NGS data using a High-performance Integrated Virtual Environment (HIVE). Database (Oxford), 2014, bau022. 24667251 10.1093/database/bau022 PMC3965850

[34] Pan Y. Karagiannis K. Zhang H. (2014) Human germline and pan-cancer variomes and their distinct functional profiles. Nucleic Acids Res., 42 (18), 11570–88. 25232094 10.1093/nar/gku772 PMC4191387

[35] Cole C. Krampis K. Karagiannis K.*.* (2014) Non-synonymous variations in cancer and their effects on the human proteome: workflow for NGS data biocuration and proteome-wide analysis of TCGA data. BMC Bioinformatics, 15, 28. 24467687 10.1186/1471-2105-15-28 PMC3916084

[36] Anders S. Huber W. (2010) Differential expression analysis for sequence count data. Genome Biol., 11, R106. 20979621 10.1186/gb-2010-11-10-r106 PMC3218662

[37] Dillies M.A. Rau A. Aubert J.*.* (2013) A comprehensive evaluation of normalization methods for Illumina high-throughput RNA sequencing data analysis. Brief. Bioinform., 14, 671–683. 22988256 10.1093/bib/bbs046

[38] R core team. (2014) A language and environment for statistical computing. R Foundation for Statistical Computing. Vienna, Austria. http://www.R-project.org/.

[39] Futreal P.A. Coin L. Marshall M. (2004) A census of human cancer genes. Nat. Rev. Cancer, 4, 177–183. 14993899 10.1038/nrc1299 PMC2665285

[40] NCBI_Resource_Coordinators. (2014) Database resources of the National Center for Biotechnology Information. Nucleic Acids Res., 42, D7–D17. 24259429 10.1093/nar/gkt1146 PMC3965057

[41] Kouprina N. Pavlicek A. Collins N.K.*.* (2005) The microcephaly ASPM gene is expressed in proliferating tissues and encodes for a mitotic spindle protein. Hum. Mol. Genet., 14, 2155–2165. 15972725 10.1093/hmg/ddi220

[42] Alsiary R. Bruning-Richardson A. Bond J. (2014) Deregulation of microcephalin and ASPM expression are correlated with epithelial ovarian cancer progression. PLoS One, 9, e97059. 24830737 10.1371/journal.pone.0097059 PMC4022499

[43] Hagemann C. Anacker J. Gerngras S. (2008) Expression analysis of the autosomal recessive primary microcephaly genes MCPH1 (microcephalin) and MCPH5 (ASPM, abnormal spindle-like, microcephaly associated) in human malignant gliomas. Oncology Rep., 20, 301–308. 18636190

[44] Bond J. Roberts E. Springell K.*.* (2005) A centrosomal mechanism involving CDK5RAP2 and CENPJ controls brain size. Nat. Genet., 37, 353–355. 15793586 10.1038/ng1539

[45] Montgomery S.H. Capellini I. Venditti C. (2011) Adaptive evolution of four microcephaly genes and the evolution of brain size in anthropoid primates. Mol. Biol. Evol., 28, 625–638. 20961963 10.1093/molbev/msq237

[46] Mulvihill M.S. Kwon Y.W. Lee S. (2012) Gremlin is overexpressed in lung adenocarcinoma and increases cell growth and proliferation in normal lung cells. PLoS One, 7, e42264. 22870311 10.1371/journal.pone.0042264 PMC3411619

[47] Weinstein J.N. Collisson E.A. Mills G.B. (2013) The Cancer Genome Atlas Pan-Cancer analysis project. Nat. Genet., 45, 1113–1120. 24071849 10.1038/ng.2764 PMC3919969

[48] Ashworth A. Hudson T.J. (2013) Genomics: comparisons across cancers. Nature, 502, 306–307. 24132284 10.1038/502306a

[49] Chew A.L. Tan W.Y. Khoo B.Y. (2013) Potential combinatorial effects of recombinant atypical chemokine receptors in breast cancer cell invasion: a research perspective. Biomed. Rep., 1, 185–192. 24648916 10.3892/br.2013.57 PMC3917100

[50] Pompella A. De Tata V. Paolicchi A. (2006) Expression of gamma-glutamyltransferase in cancer cells and its significance in drug resistance. Biochem. Pharmacol., 71, 231–238. 16303117 10.1016/j.bcp.2005.10.005

[51] Hoeller D. Hecker C.M. Dikic I. (2006) Ubiquitin and ubiquitin-like proteins in cancer pathogenesis. Nat. Rev. Cancer, 6, 776–788. 16990855 10.1038/nrc1994

[52] Ii M. Yamamoto H. Adachi Y. (2006) Role of matrix metalloproteinase-7 (matrilysin) in human cancer invasion, apoptosis, growth, and angiogenesis. Exp. Biol. Med. (Maywood), 231, 20–27. 16380641 10.1177/153537020623100103

[53] Kashiwagi K. Ishii J. Sakaeda M. (2012) Differences of molecular expression mechanisms among neural cell adhesion molecule 1, synaptophysin, and chromogranin A in lung cancer cells. Pathol. Int., 62, 232–245. 22449227 10.1111/j.1440-1827.2011.02781.x

[54] de Lange R. Dimoudis N. Weidle U.H. (2003) Identification of genes associated with enhanced metastasis of a large cell lung carcinoma cell line. Anticancer Res., 23, 187–194. 12680211

[55] Aldred M.A. Ginn-Pease M.E. Morrison C.D.*.* (2003) Caveolin-1 and caveolin-2, together with three bone morphogenetic protein-related genes, may encode novel tumor suppressors down-regulated in sporadic follicular thyroid carcinogenesis. Cancer Res., 63, 2864–2871. 12782592

[56] Hellstrom I. Raycraft J. Hayden-Ledbetter M. (2003) The HE4 (WFDC2) protein is a biomarker for ovarian carcinoma. Cancer Res., 63, 3695–3700. 12839961

[57] DeSouza L.V. Grigull J. Ghanny S. (2007) Endometrial carcinoma biomarker discovery and verification using differentially tagged clinical samples with multidimensional liquid chromatography and tandem mass spectrometry. Mol. Cell. Proteomics, 6, 1170-1182. 17374602 10.1074/mcp.M600378-MCP200

[58] Drapkin R. von Horsten H.H. Lin Y. (2005) Human epididymis protein 4 (HE4) is a secreted glycoprotein that is overexpressed by serous and endometrioid ovarian carcinomas. Cancer Res., 65, 2162–2169. 15781627 10.1158/0008-5472.CAN-04-3924

[59] Yamashita S. Tokuishi K. Hashimoto T.*.* (2011) Prognostic significance of HE4 expression in pulmonary adenocarcinoma. Tumour Biol., 32, 265–271. 20953751 10.1007/s13277-010-0118-5

[60] Chakraborty S. Kaur S. Guha S. (2012) The multifaceted roles of neutrophil gelatinase associated lipocalin (NGAL) in inflammation and cancer. Biochim. Biophys. Acta, 1826, 129–169. 22513004 10.1016/j.bbcan.2012.03.008 PMC3362670

[61] Abelson S. Shamai Y. Berger L. (2013) Niche-dependent gene expression profile of intratumoral heterogeneous ovarian cancer stem cell populations. PLoS One, 8, e83651. 24358304 10.1371/journal.pone.0083651 PMC3866276

[62] Bateman N.W. Sun M. Hood B.L. (2010) Defining central themes in breast cancer biology by differential proteomics: conserved regulation of cell spreading and focal adhesion kinase. J. Proteome Res., 9, 5311–5324. 20681588 10.1021/pr100580e

[63] Kim H. Watkinson J. Varadan V. (2010) Multi-cancer computational analysis reveals invasion-associated variant of desmoplastic reaction involving INHBA, THBS2 and COL11A1. BMC Med. Genomics, 3, 51. 21047417 10.1186/1755-8794-3-51 PMC2988703

[64] Fischer H. Stenling R. Rubio C. (2001) Colorectal carcinogenesis is associated with stromal expression of COL11A1 and COL5A2. Carcinogenesis, 22, 875–878. 11375892 10.1093/carcin/22.6.875

[65] Chong I.W. Chang M.Y. Chang H.C.*.* (2006) Great potential of a panel of multiple hMTH1, SPD, ITGA11 and COL11A1 markers for diagnosis of patients with non-small cell lung cancer. Oncol. Rep., 16, 981–988. 17016581

[66] Chapman K.B. Prendes M.J. Sternberg H. (2012) COL10A1 expression is elevated in diverse solid tumor types and is associated with tumor vasculature. Future Oncol, 8, 1031–1040. 22894674 10.2217/fon.12.79

[67] Peruzzi D. Mori F. Conforti A. (2009) MMP11: a novel target antigen for cancer immunotherapy. Clin. Cancer Res., 15, 4104–4113. 19509157 10.1158/1078-0432.CCR-08-3226

[68] Yang Y.H. Deng H. Li W.M. (2008) Identification of matrix metalloproteinase 11 as a predictive tumor marker in serum based on gene expression profiling. Clin. Cancer Res., 14, 74–81. 18172255 10.1158/1078-0432.CCR-07-1179

[69] Jung H. Lee K.P. Park S.J.*.* (2008) TMPRSS4 promotes invasion, migration and metastasis of human tumor cells by facilitating an epithelial-mesenchymal transition. Oncogene, 27, 2635–2647. 17968309 10.1038/sj.onc.1210914

[70] Sercu S. Zhang L. Merregaert J. (2008) The extracellular matrix protein 1: its molecular interaction and implication in tumor progression. Cancer Invest., 26, 375–384. 18443958 10.1080/07357900701788148

[71] Nomura H. Fujimoto N. Seiki M. (1996) Enhanced production of matrix metalloproteinases and activation of matrix metalloproteinase 2 (gelatinase A) in human gastric carcinomas. Int. J. Cancer., 69, 9–16. 8600068 10.1002/(SICI)1097-0215(19960220)69:1<9::AID-IJC3>3.0.CO;2-8

[72] Przybylowska K. Kluczna A. Zadrozny M. (2006) Polymorphisms of the promoter regions of matrix metalloproteinases genes MMP-1 and MMP-9 in breast cancer. Breast Cancer Res. Treat., 95, 65–72. 16267613 10.1007/s10549-005-9042-6

[73] Minn A.J. Gupta G.P. Siegel P.M. (2005) Genes that mediate breast cancer metastasis to lung. Nature, 436, 518–524. 16049480 10.1038/nature03799 PMC1283098

[74] Overall C.M. Kleifeld O. (2006) Tumour microenvironment—opinion: validating matrix metalloproteinases as drug targets and anti-targets for cancer therapy. Nat. Rev. Cancer, 6, 227–239. 16498445 10.1038/nrc1821

[75] Xiao T. Ying W. Li L.*.* (2005) An approach to studying lung cancer-related proteins in human blood. Mol. Cell. Proteomics, 4, 1480–1486. 15970581 10.1074/mcp.M500055-MCP200

[76] Zhu Y. Spitz M.R. Lei L. (2001) A single nucleotide polymorphism in the matrix metalloproteinase-1 promoter enhances lung cancer susceptibility. Cancer Res., 61, 7825–7829. 11691799

[77] Sunami E. Tsuno N. Osada T. (2000) MMP-1 is a prognostic marker for hematogenous metastasis of colorectal cancer. Oncologist, 5, 108–114. 10794801 10.1634/theoncologist.5-2-108

[78] Murray G.I. Duncan M.E. O'Neil P. (1996) Matrix metalloproteinase-1 is associated with poor prognosis in colorectal cancer. Nat. Med., 2, 461–462. 8597958 10.1038/nm0496–461

[79] Ye H. Yu T. Temam S. (2008) Transcriptomic dissection of tongue squamous cell carcinoma. BMC Genomics, 9, 69. 18254958 10.1186/1471-2164-9-69 PMC2262071

[80] Wang A.G. Yoon S.Y. Oh J.H.*.* (2006) Identification of intrahepatic cholangiocarcinoma related genes by comparison with normal liver tissues using expressed sequence tags. Biochem. Biophys. Res. Commun., 345, 1022–1032. 16712791 10.1016/j.bbrc.2006.04.175

[81] Bell A. Bell D. Weber R.S. (2011) CpG island methylation profiling in human salivary gland adenoid cystic carcinoma. Cancer, 117, 2898–2909. 21692051 10.1002/cncr.25818 PMC3123690

[82] Han Y.C. Zheng Z.L. Zuo Z.H. (2013) Metallothionein 1 h tumour suppressor activity in prostate cancer is mediated by euchromatin methyltransferase 1. J. Pathol., 230, 184–193. 23355073 10.1002/path.4169 PMC4080639

[83] Sakamoto L.H. DE Camargo B. Cajaiba M. (2010) MT1G hypermethylation: a potential prognostic marker for hepatoblastoma. Pediatr. Res., 67, 387–393. 20032811 10.1203/PDR.0b013e3181d01863

[84] Henrique R. Jeronimo C. Hoque M.O. (2005) MT1G hypermethylation is associated with higher tumor stage in prostate cancer. Cancer Epidemiol. Biomarkers Prev., 14, 1274–1278. 15894685 10.1158/1055-9965.EPI-04-0659

[85] Berdiel-Acer M. Cuadras D. Diaz-Maroto N.G.*.* (2014) A monotonic and prognostic genomic signature from fibroblasts for colorectal cancer initiation, progression, and metastasis. Mol. Cancer Res., 12, 1254–1266. 24829396 10.1158/1541-7786.MCR-14-0121

[86] Liu J. Li J. Li H. (2014) A comprehensive analysis of candidate genes and pathways in pancreatic cancer. Tumour Biol. doi: 10.1007/s13277-014-2787-y. 10.1007/s13277-014-2787-y 25409614

[87] Dahl E. Wiesmann F. Woenckhaus M.*.* (2007) Frequent loss of SFRP1 expression in multiple human solid tumours: association with aberrant promoter methylation in renal cell carcinoma. Oncogene, 26, 5680–5691. 17353908 10.1038/sj.onc.1210345

[88] Santana-Quintero L. Dingerdissen H. Thierry-Mieg J. (2014) HIVE-hexagon: high-performance, parallelized sequence alignment for next-generation sequencing data analysis. PLoS One, 9, e99033. 24918764 10.1371/journal.pone.0099033 PMC4053384

[89] Ellis M.J. Gillette M. Carr S.A.*.* (2013) Connecting genomic alterations to cancer biology with proteomics: the NCI Clinical Proteomic Tumor Analysis Consortium. Cancer Discov., 3, 1108–1112. 24124232 10.1158/2159-8290.CD-13-0219 PMC3800055

